# Epigenetic repression of ribosomal RNA transcription by ROCK-dependent aberrant cytoskeletal organization

**DOI:** 10.1038/srep28685

**Published:** 2016-06-28

**Authors:** Tse-Hsiang Wu, Yuan-Yeh Kuo, Hsiao-Hui Lee, Jean-Cheng Kuo, Meng-Hsin Ou, Zee-Fen Chang

**Affiliations:** 1 Institute of Biochemistry and Molecular Biology, National Taiwan University, No. 1, Section 1, Jen-Ai Road, Taipei, Taiwan 100, R.O.C; 2 Institute of Oncology, College of Medicine, National Taiwan University, No. 1, Section 1, Jen-Ai Road, Taipei 100, Taiwan, R.O.C; 3 Department of Life Sciences and Institute of Genome Sciences, National Yang-Ming University, No.155, Sec.2, Linong Street, Taipei, 112, Taiwan, R.O.C; 4 Institute of Biochemistry and Molecular Biology, National Yang-Ming University, No.155, Sec.2, Linong Street, Taipei, 112, Taiwan, R.O.C

## Abstract

It is known that ribosomal RNA (rRNA) synthesis is regulated by cellular energy and proliferation status. In this study, we investigated rRNA gene transcription in response to cytoskeletal stress. Our data revealed that the cell shape constrained by isotropic but not elongated micropatterns in HeLa cells led to a significant reduction in rRNA transcription dependent on ROCK. Expression of a dominant-active form of ROCK also repressed rRNA transcription. Isotropic constraint and ROCK over-activation led to different types of aberrant F-actin organization, but their suppression effects on rRNA transcription were similarly reversed by inhibition of histone deacetylase (HDAC) or overexpression of a dominant negative form of Nesprin, which shields the signal transmitted from actin filament to the nuclear interior. We further showed that the binding of HDAC1 to the active fraction of rDNA genes is increased by ROCK over-activation, thus reducing H3K9/14 acetylation and suppressing transcription. Our results demonstrate an epigenetic control of active rDNA genes that represses rRNA transcription in response to the cytoskeletal stress.

Actomyosin-mediated contractility regulates cytoskeleton organization, which is essential for many biological processes including cell division, migration and morphogenesis^1^. Compelling evidence has shown that different physical cues influence transcriptional regulation to dictate cell behaviors and differentiation lineages through cytoskeletal signals^2^,^3^,^4^,^5^,^6^. In this study, we investigated whether ribosomal RNA (rRNA) transcription is regulated by cytoskeletal changes. We chose ribosomal RNA (rRNA) genes for this study, because rRNA transcription represents over 60% of total transcription and is highly sensitive to a variety of stresses such as energy deficiency and DNA damage^7^. Moreover, it has been shown that extracellular force application causes redistribution of nucleoli where rDNA genes are organized^8^.

Transcription of rDNA genes in the nucleolus generates 45S pre-RNAs, which are subsequently cleaved and processed into the 28S, 18S, and 5.8S rRNAs. These rRNAs are then packaged for ribosome biogenesis^9^. Within the human genome, there are over 400 copies of the rRNA genes clustered into nucleolar organizer regions (NOR) in euchromatin and heterochromatin states^10^. For actively transcribed rDNA genes, acetylated H3K9/14 is associated with the promoter, and RNA Polymerase I (pol I) is recruited to the promoter through the cooperation between upstream binding factor (UBF) and SL1 complex for transcription initiation and elongation^11^. The rDNA gene copies in euchromatic state are associated with the UBF; they can either be actively transcribed or transcriptionally repressed, while genes in heterochromatin state lack UBF association and are epigenetically silenced^12^. Thus, rRNA transcription is regulated by different layers of epigenetic control.

It is well established that active ROCK phosphorylates myosin light chain (MLC) and inactivates MLC phosphatase, thus increasing myosin II ATPase activity to generate actomyosin-mediated contractility^13^,^14^. The kinase activity of ROCK is negatively regulated by its intramolecular auto-inhibition^15^, which is relieved by RhoA binding^16^. Compelling evidence has indicated that ROCK-mediated myosin II activity regulates cell shape, motility and differentiation^17^. In this study, we used fibronectin (FN) micropattern to constrain cell shapes and overexpression of a dominant active form of ROCK, which is deleted of autoinhibitory domain^18^, to deregulate myosin II-mediated force. Ribosomal RNA transcription at nucleolar sites in response to these alterations in cytoskeleton was determined, and the mechanism responsible for the response was further explored.

## Results

### Isotropic constraint represses rRNA transcription dependent on ROCK and histone deacetylation

**Figure 1:**
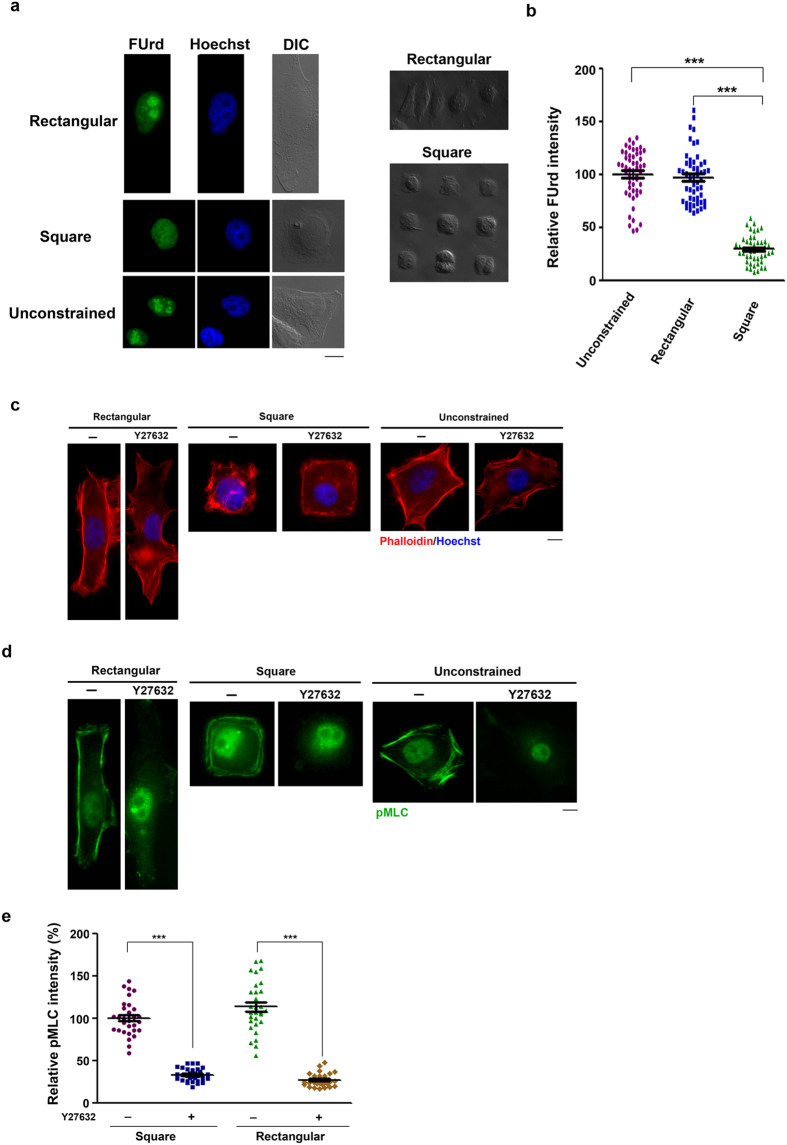
Square-micropatterned constraint leads to rRNA transcription repression and aberrant ROCK-dependent F-actin organization. (**a**) Effect of cell shape on FUrd incorporation in nucleoli. HeLa cells were plated onto 18.5 × 55.4 μm (1024 μm^2^) FN-coated rectangular and 32 × 32 μm (1024 μm^2^) FN-coated square micropatterns. In parallel, cells were plated onto regular culture dish. After 6 h, cells were pulse-labeled with FUrd for 20 min and fixed for nuclear staining by Hoechst 33342 and IF staining using BrdU antibody to indicate FUrd incroporation. Representative images of IF of FUrd incorporation/nuclear staining/DIC in cells without geometry constraint (bottom panel) and with micropattern restricted (upper and middle panel) are shown; scale bar, 10 μm. (**b**) The relative FUrd labeling intensity in cells. The mean of anti-BrdU antibody fluorescence in unconstrained cells was set to 100%. Values represent mean ± SEM, (total n > 50), ***P < 0.001. (**c**,**d**) Analysis of F-actin and pMLC distribution. HeLa cells as indicated were treated with and without Y27632 (10 μM) for 4 h, followed by fixation for (**c**) Phalloidin and (**d**) pMLC IF staining. Cells were co-stained with Hoechst 33342; scale bar, 10 μm. (**e**) The quantitation of ROCK-dependent pMLC intensity in peripheral region. Data are expressed relative to the intensity in untreated round pattern cells.

HeLa cells were plated onto FN-micropatterned substrates. Micropatterns were in square (1:1) or rectangular (1:3) shape with the same area (1024 μm^2^). This size of micropattern area is smaller than that of unconstrained cell spreading onto regular dish. The ongoing rRNA transcription was assessed by fluorouridine (FUrd) incorporation at the nucleolar site using BrdU antibody for immunofluorescence (IF) staining^19^,^20^. Cells plated onto rectangular micropattern and the regular culture dish had similar extent of FUrd incorporation at nucleolar sites. In square-constrained cells, FUrd incorporation at nucleolus sites was significantly reduced as compared to those in rectangular-constrained and unconstrained cells (Fig. 1a,b). In this experiment, there were two cells staying on one square pattern, and these cells had higher levels of total intensity of nucleolar FUrd incorporation than those single cells on one square-island (Fig. S1). Clearly, the geometry by isotropic retraction represses rRNA transcription.

It is known that the molecular interactions between F-actin and ROCK-mediated non-muscle myosin II controls cell shapes^1^. Cells constrained on square, rectanglar shapes and without constraint on a regular dish were treated with and without Y27632, a specific ROCK inhibitor^21^,^22^, to compare ROCK-dependent F-actin organization. Phalloidin staining showed that cells on rectangular islands and the regular culture dish had long F-actin filaments aligned in the cortical region. Distinctly, square-constrained cells had many star-like F-actin patches in short length all over the cell body (Fig. 1c). Y27632 treatment abolished major F-actin organization patterns in all cells. We also compared ROCK-dependent MLC phosphorylation (pMLC) in these cells. The IF staining showed that pMLC mainly distributed along the peripheral region in square-, rectangular-constrained and unconstrained cells (Fig. 1d). By quantitation, we did not find a significant difference in Y27632-sensitive pMLC intensity in rectangular and square cells (Fig. 1e). Therefore, rRNA transcription is correlated with the changes in F-actin organization rather than the overall intensity of myosin II activity.

**Figure 2:**
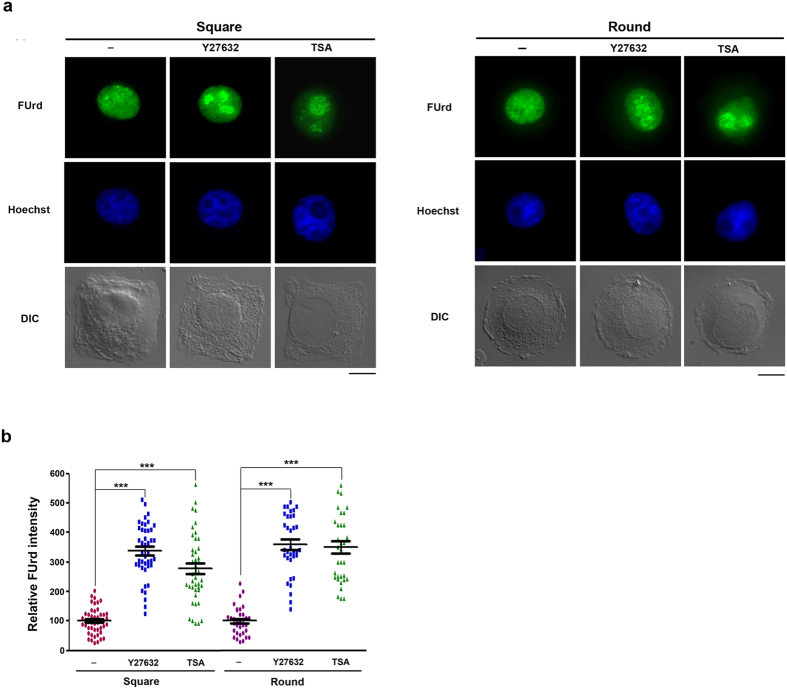
Inhibition of ROCK or HDAC restores rRNA transcription in square- and round-micropatterned cells. HeLa cells were plated on FN-micropatterns in square (32 × 32 μm) and round (r = 18.06 μm) shapes and treated with 10 μM Y27632 and TSA (1 μg/ml) as indicated. After 6 h, cells were pulse-labeled with FUrd and fixed as described in the legend to Fig. 1a. (**a**,**b**) Representative images of FUrd incorporation IF/nuclear staining/DIC restricted in square and round shapes (scale bar, 10 μm). (**b**) Relative FUrd labeling intensity in the cells. Data are expressed relative to the intensity in untreated cells. Values represent mean ± SEM (total n = 42–52 cells), ***P < 0.001.

Since F-actin organization is ROCK-dependent, the effect of Y27632 treatment on rRNA transcription in square- or round-constrained cells was examined. We found that Y27632 treatment significantly increased the levels of nucleolar FUrd incorporation in these cells (Fig. 2a,b). We also treated these cells with trichostatin (TSA), a general inhibitor of HDACs^23^. Like Y27632 treatment, nucleolar FUrd incorporation was also markedly increased by TSA treatment. Thus, ROCK-dependent F-actin organization in isotropic constrained cells is sufficient to cause rRNA transcription repression via HDAC activity.

### ROCK(CAT) expression causes stress fiber formation and HDAC-dependent suppression of rRNA transcription

**Figure 3:**
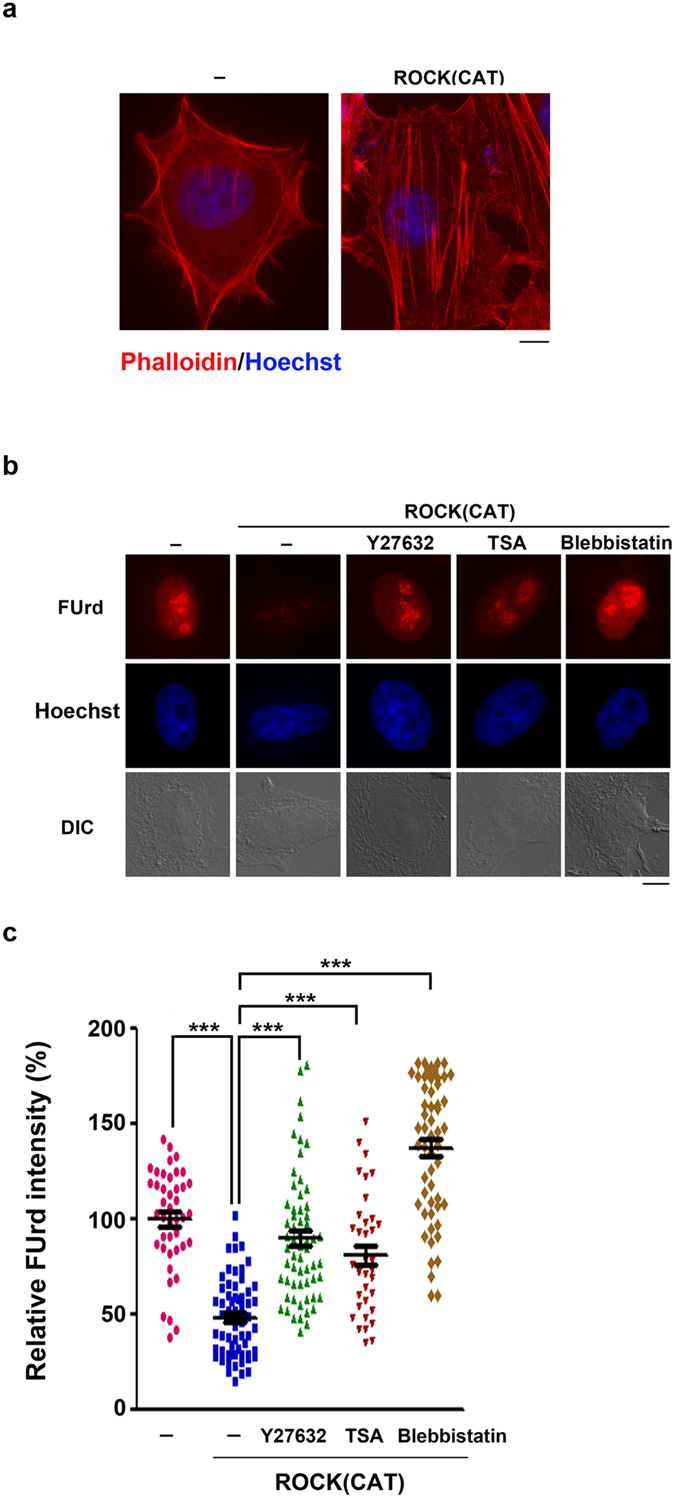
ROCK(CAT) expression leads to stress fiber formation and rRNA repression via histone deacetylation. Expression vector of control or ROCK (CAT) was co-transfected with pGFP-C1 (ratio of 3:1 in DNA amount) to HeLa cells for 10 h. (**a**) Cells were fixed for nuclear staining by Hoechst 33342 and Phalloidin. Representative images of green-positive cells are shown; scale bar, 10 μm. (**b**) Cell were treated with Y27632(10 μM), TSA (1 μg/ml) and Blebbistatin (50 μM) for 5 h as indicated, followed by pulse-labeling with FUrd and fixed as described in the legend to Fig. 1a. Representative images of IF of FUrd incorporation/nuclear staining are shown; scale bar, 10 μm. (**c**) Relative FUrd labeling intensity in cells. Data are expressed relative to the intensity of anti-BrdU IF in control cells. Values represent mean ± SEM (total n = 35‒61 cells), ***P < 0.001.

We also detached HeLa cells by trypsinization to cause cell rounding. Under this condition, rRNA transcription was also switched-off. Either inhibition of ROCK or myosin II ATPase activity by Y27632 or blebbstatin^24^, respectively, was sufficient to restore rRNA transcription (Fig. S2). The kinase activity of ROCK is regulated by auto-inhibition, which is relieved by RhoA binding to drive myosin II activation^18^. Our previous studies have shown that RhoA binding affinity of ROCK is reciprocally regulated by Src kinase and Shp2 phosphatase^25^,^26^. Therefore, ROCK-mediated myosin II activity is finely tuned in a cell to coordinate physiological processes. We further overexpressed ROCK(CAT), a constitutively active form of ROCK deleted of its autoinhibitory domain^18^, to test whether the loss of negative regulation of ROCK is sufficient to repress rRNA transcription in cells. Phalloidin staining showed that overexpression of ROCK(CAT) generates stress fibers in HeLa cells (Fig. 3a). FUrd incorporation assay indicated that overexpression of ROCK(CAT) reduced rRNA transcription in nucleoli. Treatment of cells with blebbistatin or Y27632 reversed the repression (Fig. 3b,c). In addition, TSA treatment also prevented ROCK(CAT)-induced repression of nucleolar FUrd incorporation. These results indicate that over-activation of myosin II activity that generates abnormal stress fibers is also sufficient to cause HDAC-mediated rRNA repression.

### The reversal of ROCK-dependent repression of rRNA transcription by disrupting the links between nucleoskeleton and cytoskeleton

**Figure 4:**
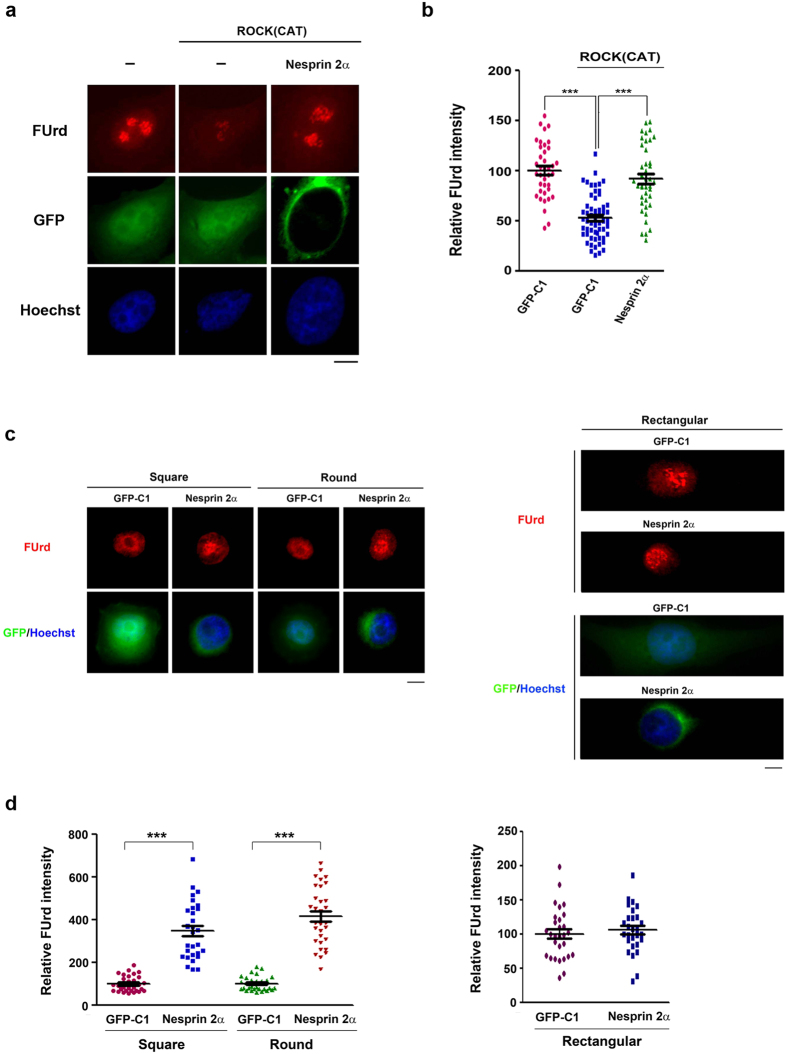
Nesprin2α overexpression overcomes ROCK(CAT)- or isotropic constraint-induced repression of rRNA transcription. (**a**,**b**) Effect of Nesprin 2α overexpression on ROCK(CAT)-induced repression. Expression vector of ROCK (CAT) was transfected to HeLa cells in the presence of Nesprin 2α-GFP or GPF-C1expression vector for 10 h. Cells were pulse-labeled with FUrd and fixed as described in the legend to Fig. 1 (**a**) Representative images of FUrd IF/nuclear staining; scale bar, 10 μm. (**b**) Relative FUrd labeling intensity at nucleolar sites in cells. Dara are expressed relative to the intensity of anti-BrdU IF in untransfected cells. Values represent mean ± SEM (total n > 50), ***P < 0.001. (**c**,**d**) Effect of Nesprin 2α overepxression on rRNA repression by isotropic constraint. HeLa cells were transfected with Nesprin 2α-GFP or GPF-C1expression vector for 10 h. Cells were plated onto in rectangular, square, and round FN-micropattern with the same surface area (1024 μm^2^). After 6 h, Cells were pulse-labeled with FUrd and fixed for IF as described in the legend to Fig. 1a. (**c**) Representative images of IF/nuclear staining; scale bar, 10 μm. (**d**) The relative FUrd labeling intensity at nucleolar sites in cells. Data are shown relative to the intensity of anti-BrdU IF in pGPF-C1 transfected cells. Values represent mean ± SEM (total n = 30–36 cells), ***P < 0.001.

It is established that nucleoskeleton is connected with cytoskeleton by the LINC complex (Linker of Nucleoskeleton and Cytoskeleton), which is composed of nesprin and SUN proteins^27^,^28^. Nesprin proteins cross the outer nuclear membrane to interact with cytoskeletal elements such as actin-filaments, and their KASH domains in inner membrane interact with SUN proteins which are associated with nuclear lamins and chromatin^29^. Through this linkage, the LINC complex has a critical function in sensing a variety of mechanical stimuli. Four nesprin proteins are identified. Nesprin-1 and -2 contain an N-terminal actin-binding domain^30^. Nesprin-2α lacks the actin-binding domain but still retains the KASH domain for interaction with SUN proteins^31^. Therefore, overexpression of Nesprin-2α is able to disrupt the signal transmitted from actin filaments to nuclear interior^32^. We then tested the effect of Nesprin-2α overexpression on rRNA transcription in cells with ROCK(CAT) overexpression or constrained by square, round and rectangular micropatterns. The results showed that co-expression of Nesprin 2α overcame the inhibitory effect of ROCK(CAT) on rRNA repression (Fig. 4a). Nesprin 2α overexpression also significantly reversed the transcriptional repression in square- and round-constrained cells, while having little effect on the elongated cells (Fig. 4b). Taken together, these data suggest that F-actin disorganization by enforcing either isotropic constraint or ROCK over-activation transmits a signal through the LINC complex to repress rRNA transcription by histone deacetylation.

### ROCK(CAT) induction reduces Pol I recruitment to rDNA genes without affecting UBF binding

**Figure 5:**
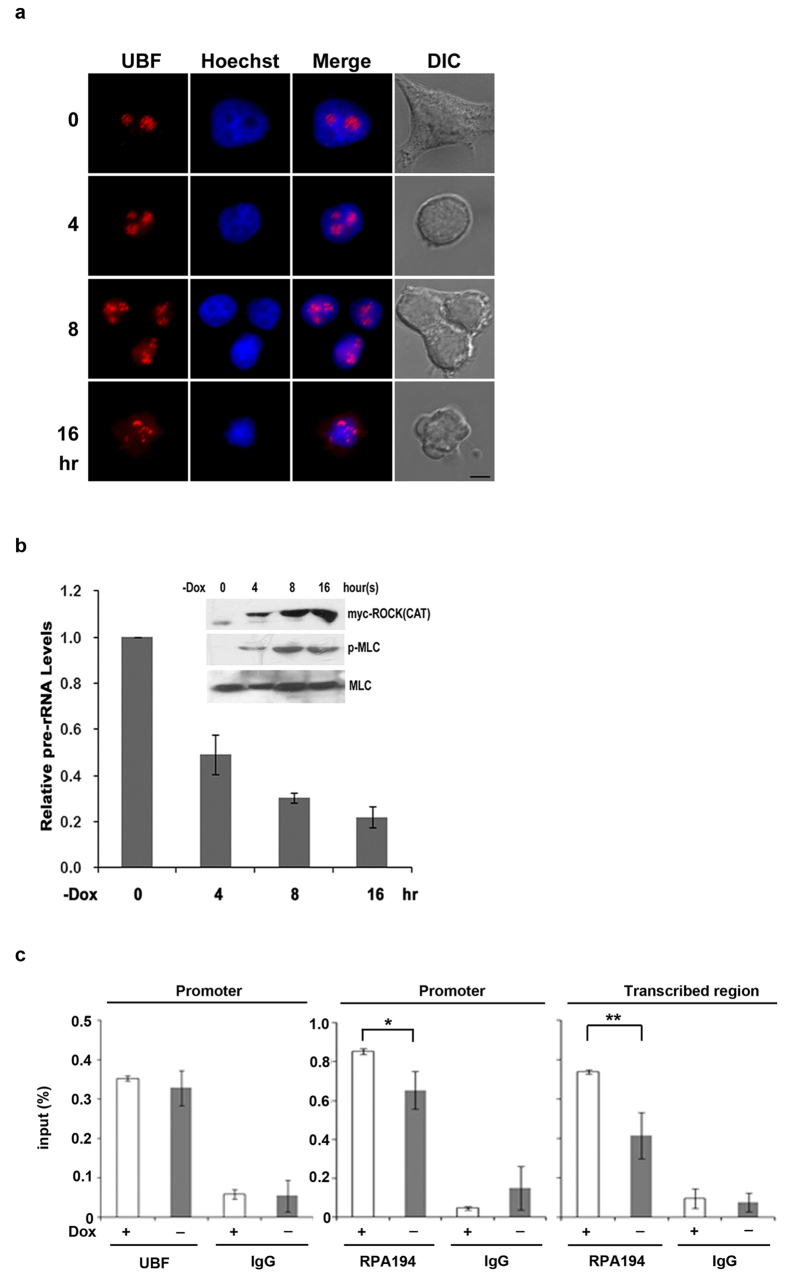
Induction of ROCK(CAT) increases MLC phosphorylation and reduces Pol I recruitment to rDNA genes without affecting UBF binding. HEK293T cells were transfected with pUHD10.3-myc-ROCK (CAT) in the presence of doxycycline (20 ng/ml). For induction, cells were trypsinized and washed with PBS for 3 times prior to incubation in the medium without doxycycline for induction. (**a**) Along the withdrawal of doxycycline (-Dox) at the indicated time, cells were stained with UBF antibody (red) and Hoechst (blue); scale bar, 10 μm. (**b**) Cells were harvested for RT-qPCR analysis using primers specific for 45S pre-rRNA and for Western blotting (inlet) with myc, phospho-MLC (p-MLC), or MLC antibody. (**c**) After ROCK (CAT) induction for 6 h, cells were treated with formaldehyde for ChIP analysis using antibody of UBF, RPA194 and IgG. PCR data from ChIP were divided by total input. Values represent mean ± S.D. (*n* = 3), *P < 0.05; **P < 0.01.

Considering that prolonged expression of ROCK (CAT) causes nucleolar dispersion, we further expressed ROCK (CAT) under the tet-off promoter in HEK293T cells. The IF staining of UBF was performed to examine the nucleolar organization during doxycycline withdrawal for ROCK(CAT) induction. At 4 h, cells became rounded with nucleoli still in organized form. At 16 h of induction, nucleoli became dispersed (Fig. 5a). We also harvested cells to analyze time-dependent change of new synthesis of 45S pre-rRNA and MLC phosphorylation during ROCK(CAT) induction. We found the synthesis of 45S pre-rRNA reduced 50% at 4 h followed by a further reduction after longer ROCK (CAT) induction (Fig. 5b). Apparently, pre-rRNA repression by ROCK(CAT) induction occurred before nucleolar disassembly.

We then used this inducible system to assess whether the binding of UBF, a general transcription factor, and RPA194, a subunit of pol I, to rDNA genes by ChIP assay. The results showed that ROCK(CAT) induction decreased RPA194 binding to the promoter and transcribed regions without affecting the binding of UBF to the promoter of rDNA genes (Fig. 5c).

### ROCK(CAT) induction increases HDAC1-mediated H3K9/14 deacetylation to repress rRNA transcription

**Figure 6:**
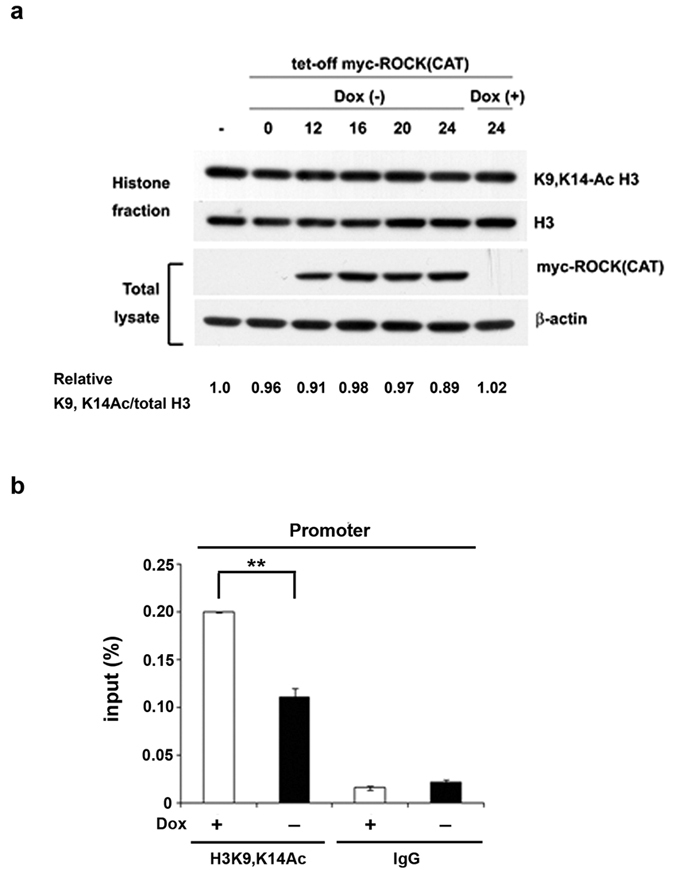
Induction of ROCK(CAT) reduces K9/14 acetylation of H3 associated with the rDNA gene promoter. HEK293T cells with ROCK (CAT) induction by Dox withdrawal were harvested for (**a**) Western blot analysis of histone fractionation and total lysates. The relative intensity of K9/14 acetylation of H3 normalized by total amount of H3 in histone fraction is shown below. (**b**) ChIP analysis using antibodies against acetylated H3K9/14 and UBF as indicated. ChIP samples were applied to Q-PCR for quantification. Data represent mean ± S.D. (*n* = 3), **P < 0.01.

**Figure 7:**
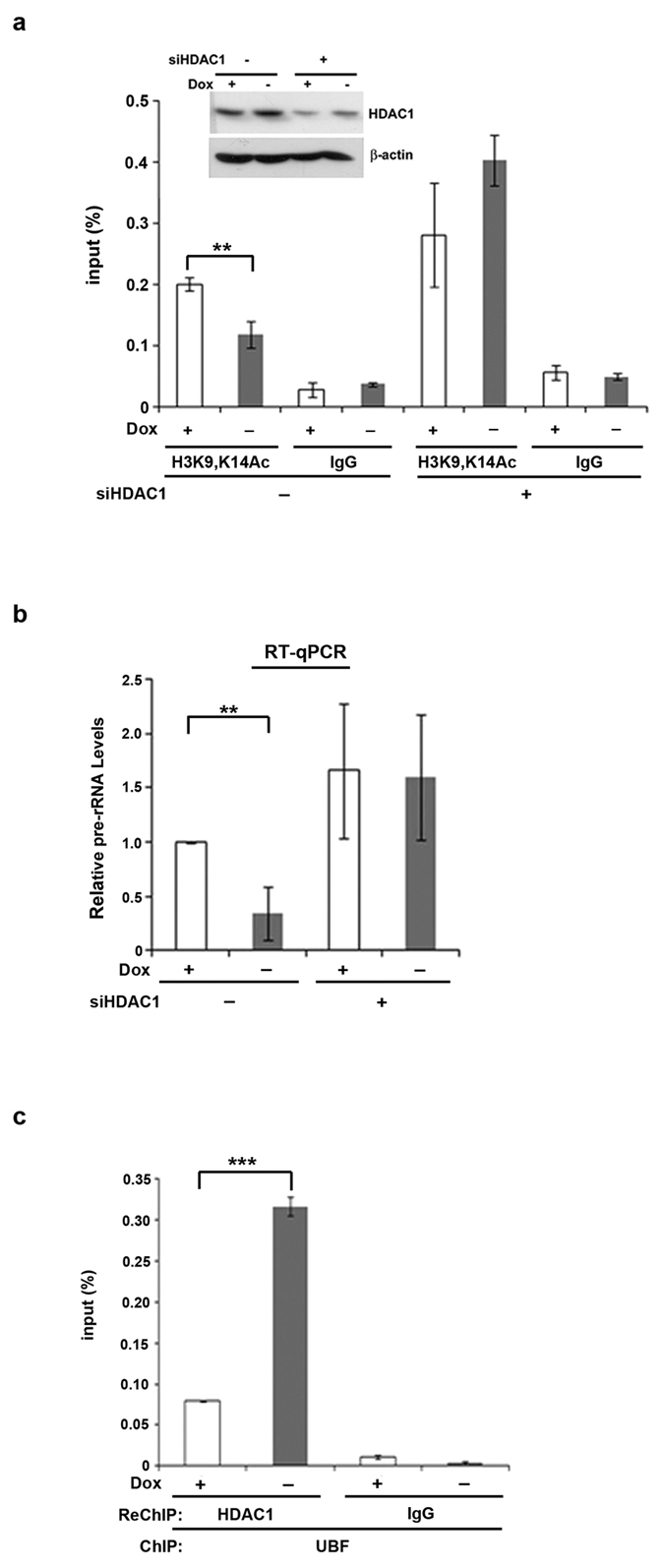
Induction of ROCK(CAT) causes HDAC1-mediated H3K9/14 deacetylation to repress rRNA transcription. HEK293T cells were transfected with HDAC1 siRNA for 2 days, followed by on or off ROCK (CAT) induction. (**a**) After 6 h, cells were subjected to the qChIP assay using an H3K9/14Ac antibody. The immunoblot shows HDAC1 knockdown. Data represent mean ± S.D. (*n* = 3), **P < 0.01. (**b**) Analysis of pre-rRNA level in HEK293T cells. Data represent mean ± S.D. (*n* = 3), **P < 0.01. (**c**) After 6 h, cells were subjected to the qChIP assay using a UBF antibody, followed by reChIP with an HDAC1 antibody for qPCR of the rDNA promoter. Data represent mean ± S.D. (*n* = 3), ***P < 0.001.

It has been reported that global H3K9/14 acetylation is significantly decreased by culturing mammary epithelial cells in 3D culture^33^. Here, we did not find a reduction in global H3K9/14 acetylation in response to ROCK(CAT) induction (Fig. 6a). Since H3K9/14 acetylation is a chromatin mark in active rDNA transcription, we wanted to know whether H3K9/14 acetylation associated with rDNA genes is altered in a ROCK(CAT)-responsive manner. At 6 h after ROCK (CAT) induction, H3K9/14 acetylation associated with the rDNA promoter was clearly decreased (Fig. 6b). We further transfected cells with HDAC1 siRNA to decrease HDAC1 expression. The results of ChIP analysis and RT-qPCR of 45S pre-rRNA showed that HDAC1 knockdown increased the level of acetylated H3K9/14 bound with the promoter (Fig. 7a) and restored pre-rRNA synthesis in cells with ROCK(CAT) induction (Fig. 7b). Next, we tested whether there is an increase in HDAC1 occupancy on the rRNA genes in euchromatic state. Since ROCK(CAT) induction does not affect UBF binding with euchromatic rDNA genes, we performed ChIP by UBF and reChIP by HDAC1 antibody. The results showed that HDAC1 association with the promoter of euchromatic rDNA genes was increased by ROCK (CAT) induction (Fig. 7c). Thus, ROCK(CAT) expression increases HDAC1 binding to the promoter in active fraction of rDNA genes, resulting in H3K9/14 deacetylation and transcriptional repression of rRNA.

## Discussion

A cell is integrated by a mechanical network connecting the nucleus, the cytoskeleton with extracellular matrix in adhesion^29^. In this study, we found that F-actin organization by isotropic constraint or overexpression of ROCK(CAT) in HeLa cells leads to rRNA transcription repression. Inhibition of HDAC activity or blocking the links between nucleoskeleton and cytoskelen is sufficient to restore rRNA transcription in these cells, suggesting that aberrant F-actin stress can transduce a signal to repress rDNA gene through histone deacetylation. Our mechanistic investigation further indicated that over-activation of ROCK increases HDAC1 binding to rDNA gene. As a result, pol I recruitment and H3K9/14 acetylation are reduced, thus repressing pol I-mediated rRNA transcription. Given that HDAC1 knockdown abrogates the repression effect of ROCK(CAT) induction, our data suggest a stimulation of HDAC1 binding to active rDNA genes by the cytoskeletal stress, leading to rRNA repression.

It has been shown that inhibition of pol I results in nucleoli disassembly^34^. Despite of the differences in cell and nuclear morphology due to various micropatterns or ROCK(CAT) expression, nucleoli remained intact in these cells. Since inhibition of ROCK or HDAC is sufficient to restore rRNA transcription in cells constrained in square-, round-micropattern or with induction of ROCK(CAT), it is apparent that the transcriptional repression of rDNA genes is not a result of the loss of functional pol I machineries or global architectural defect in nucleoli. It is known that UBF binding is not only essential for transcription initiation, but also has a function in the organization of active rDNA genes. Given that UBF binding to rDNA genes remains unchanged in response to ROCK(CAT) induction, the repression is specifically controlled at the level of HDAC1 binding to active rDNA genes. Relevant to our observation, it has been reported that global H3K9 acetylation is reduced in round epithelial cell when cultured in lamin-free 3D condition^33^. In our ROCK(CAT) induction system, we did not observe a global change in H3K9 acetylation. Therefore, it is more likely that the open chromatin structure of rDNA genes is highly accessible by HDAC1 in response to the physical stress signal.

In the cells with ROCK(CAT) expression or isotropic constraint, rRNA transcription is restored by overexpression of Nesprin 2α, which disrupts the connectivity function of LINC complex due to the deficiency in binding with actin filament. Therefore, its reversal effect on rRNA transcript in these cells is likely through abrogating the stress signal transmitted from aberrant F-actin structures. This also implies that the epigenetic control of the active fraction of rDNA genes in nucleoli is linked to the cytoskeleton.

As ribosome biogenesis is essential for protein synthesis that demands high energy supply, the transcription rate of rDNA genes is very sensitive to extra- or intracellular stresses. For examples, UV, IR irradiation or mTOR inhibition down-regulates rRNA transcription by abrogating Pol I initiation complex formation via different signal pathways^35^,^36^,^37^. Glucose deprivation has been shown to induce a heterochromatin status of rDNA loci through an epigenetic silencing complex, thus suppressing rRNA transcription^38^. In summary, our data highlight an epigenetic regulation of rRNA transcription sensitive to the signal from the cytoskeletal stress through the connectivity across nuclear envelope.

## Materials and Methods

### Micropattern preparation

A thick layer of degassed PDMS mixture was spread onto 22 × 22 mm2 coverslips by swienco-PM490 instrument and cured them for 5 minutes in a 110 °C oven. The coverslips were irradiated by UV ocleaner (Jelight) for 1 minute before stamping. Wafers stamps with the desired designs were coated with fibronectin (50 mg/ml) at 37 °C for 1 h. The FN-coated stamps was put onto PDMS-containing coverslips for 5 minutes, followed by incubation with 0.2% blocking solution blocking (Pluronic® F-127, sigma P2443-250G) at room temperature for 2 h.

### Plasmid constructs and transfection

DNA fragment of myc-ROCK (CAT), spanning 6-553 amino acid, was subcloned to pUHD10.3. For on and off expression of ROCK (CAT), cells were transfected with a mixture of pUHD10.3-myc-ROCK (CAT) and pUHD15.1 (with a ratio of 2:1) by Nanofectin (PAA Laboratories, Pasching, Austria) and cultivated in medium containing 20 ng/ml doxycycline (Sigma).

### Antibodies and reagents

H3K9/14ac and H3 antibodies were purchased from Upstate Biotechnology. Anti-UBF and anti-HA antibodies were from Santa Cruz. Anti-phospho-MLC2 antibody was obtained from Cell Signaling Technology. Anti-MLC, anti-BrU, anti-β-actin antibodies, doxycycline, and trichostatin A (TSA) were obtained from Sigma-Aldrich. Anti-myc antibody was purified from hybridoma clone 9E10. HDAC1 antibody was from Abcam. Y27632 and Blebbistatin were from Merck Biosciences.

### FUrd incorporation analysis and quantification

Cells seeded on coverslips were labeled with 2 mM fluorouridine (FUrd) for 20 min at 37 °C. Cells were then fixed by 3.7% *para*-formaldehyde, permeablized with methanol at −20 °C, and incubated with a BrU antibody (Sigma-Aldrich), followed by incubation with TRITC- or FITC-conjugated secondary antibody. The coverslips were examined by a fluorescence microscope (Carl Zeiss, AxioObserver A1). The intensity of FUrd incorporation at nucleolus sites in each cell was quantified using the ImageJ software. A two-tailed Student’s t test was used to assess IF intensity at nucleoli in each cells (***P < 0.001)

### F-actin staining and Immunofluorescence analysis of UBF and pMLC

For F-actin analysis, cells were fixed with 4% paraformaldehyde in PBS at room temperature for 10 min, followed by staining with Palloidin-Rhodamine (Invitrogen) at 1:400 dilution. For UBF and pMLC IF staining, cells were fixed with 4% paraformaldehyde, permeablized with 0.3% Triton X-100 (TBST), and incubated with antibodies against UBF and pMLC, followed by incubation with TRITC-rabbit and FITC-rabbit conjugated secondary antibodies, respectively, with Hoechst 33342. All images were acquired in a Carl Zeiss AxioObserver A1 with the 63× oil objective using a cooled CDD camera operated by AxioVision image software.

### RNA Isolation and RT-qPCR

Total RNA was isolated from cells by using RNAzol B reagent (Tel-Test). cDNA was synthesized using ImProm-II reverse transcriptase (Promega). Real-time quantitative PCR analysis was performed using Stepone Real-Time PCR system (Applied Biosystems) and Fast SYBER Green Master Mix (Applied Biosystems). 45S pre-rRNA was normalized to 18S rRNA. The primer sequences were as follows: human 45S pre-rRNA sense primer 5′-CCTGCTGTTCTCTCGCGCGTCCGAG-3′, antisense primer 5′-AACGCCTGACACGCACGGCACGGAG-3′; human 18S rRNA sense primer 5′-CGACGACCCATTCGAACGTCT-3′, antisense primer 5′-CTCTCCGGAATCGAACCCTGA-3′.

### Chromatin immunoprecipitation (ChIP) and ReChIP assays

In brief, cells (5 × 10^6^) were treated with formaldehyde at a final concentration of 1% for 10 min at room temperature followed by glycine quench. DNA were fragmented to averaging <1 kb for antibody immunoprecipitation. After washing, elution, deproteination, and heating, immunoprecipitated DNA was extracted and ethanol precipitated. DNA was resuspended in 50 μl of Tris-EDTA buffer. For chromatin reimmunoprecipitation (reChIP), chromatin cross-linked to the immuno-complex was eluted by incubation with 50 μl of dithiothreitol (10 mM) at 37 °C for 30 min. After centrifugation, the supernatant was diluted with 20x volume of buffer [20 mM Tris-HCl, (pH 8.1), 150 mM NaCl, 2 mM EDTA, 1% Triton X-100] and was subjected to the ChIP procedure as described above. PCR was applied to the immunoprecipitated DNA with the following thermal cycling program: 30 s at 94 °C, 30 s at 55 °C and 1 min at 72 °C (30 cycles for first ChIP and 35 cycles for reChIP), followed by a extension time at 72 °C for 5 min. PCR products were analyzed on 2.0% agarose gel and visualized by ethidium bromide staining. Immunoprecipitated DNA was further quantified by qPCR. The ratio of rDNA in the immunoprecipitates versus rDNA in the input chromatin was normalized to that from control reactions. Real-time quantitative PCR analysis was performed using Stepone Real-Time PCR system (Applied Biosystems) and Fast SYBER Green Master Mix (Applied Biosystems). The primer sequences were as follows: human rDNA promoter sense primer 5′-CGATGGTGGCGTTTTTGG-3′, antisense primer 5′-CCGACTCGGAGCGAAAGATA-3′. The 18S rRNA primer pair was used for amplification of the transcribed region.

### Histone preparation

Nuclei pellet was prepared and extracted with 0.2N H_2_SO_4_ on ice for 1 h followed by centrifugation at 12,000 rpm for 10 minutes at 4 °C. The supernatant was precipitated by 20% Trichloroacetic acid (TCA). After centrifugation at 12,000 rpm for 10 minutes at 4 °C, histone pellet was obtained.

### Statistical analysis of qChIP and RT-PCR data

qChIP and RT-qPCR data were analyzed from three independent experiments and expressed by mean values of 95% confidence interval using a two-tailed Student’s *t*-test. Data represent mean ± S.D. (*n* = 3), *P < 0.05, **P < 0.01, ***P < 0.001.

### RNA interference experiments

siRNA targeting human *HDAC1* was purchased from Invitrogen. The siRNA sequences were as follows. *siHDAC1* sense: CAGCGACUGUUUGAGAACCTT, antisense: GGUUCUCAAACAGUCGCUGTT.

## Additional Information

**How to cite this article**: Wu, T.-H. *et al*. Epigenetic repression of ribosomal RNA transcription by ROCK-dependent aberrant cytoskeletal organization. *Sci. Rep.* **6**, 28685; doi: 10.1038/srep28685 (2016).

## Supplementary Material

Supplementary Information
