# Supplementary material for: Epigenetic repression of ribosomal RNA transcription by ROCK-dependent aberrant cytoskeletal organization
Source: Sci Rep. 2016 Jun 28;6:28685. doi: 10.1038/srep28685 (PMC4923894; doi:10.1038/srep28685)
Supplement: Supplementary Information [file srep28685-s1.doc]

**Title:** Epigenetic repression of ribosomal RNA transcription by ROCK-dependent

aberrant cytoskeletal organization

**Authors:** Tse-Hsiang Wu^1^, Yuan-Yeh Kuo^2^, Hsaio-Hui Lee^3^, Jean-Cheng Kuo^4^,

Meng-Hsin Ou^4^ and Zee-Fen Chang^4^

^1^Institute of Biochemistry and Molecular Biology,^2^Institute of Oncology, College of Medicine, National Taiwan University, No. 1, Section 1, Jen-Ai Road, Taipei, Taiwan, R.O.C., ^3^Department of Life Sciences and Institute of Genome Sciences, ^4^Institute of Biochemistry and Molecular Biology, National Yang-Ming University, No.155, Sec.2, Linong Street, Taipei, 112 Taiwan

corresponding author, [zfchang@ym.edu.tw](mailto:ZFCHANG@ym.edu.tw)

**Supplementary Figure 1**

**
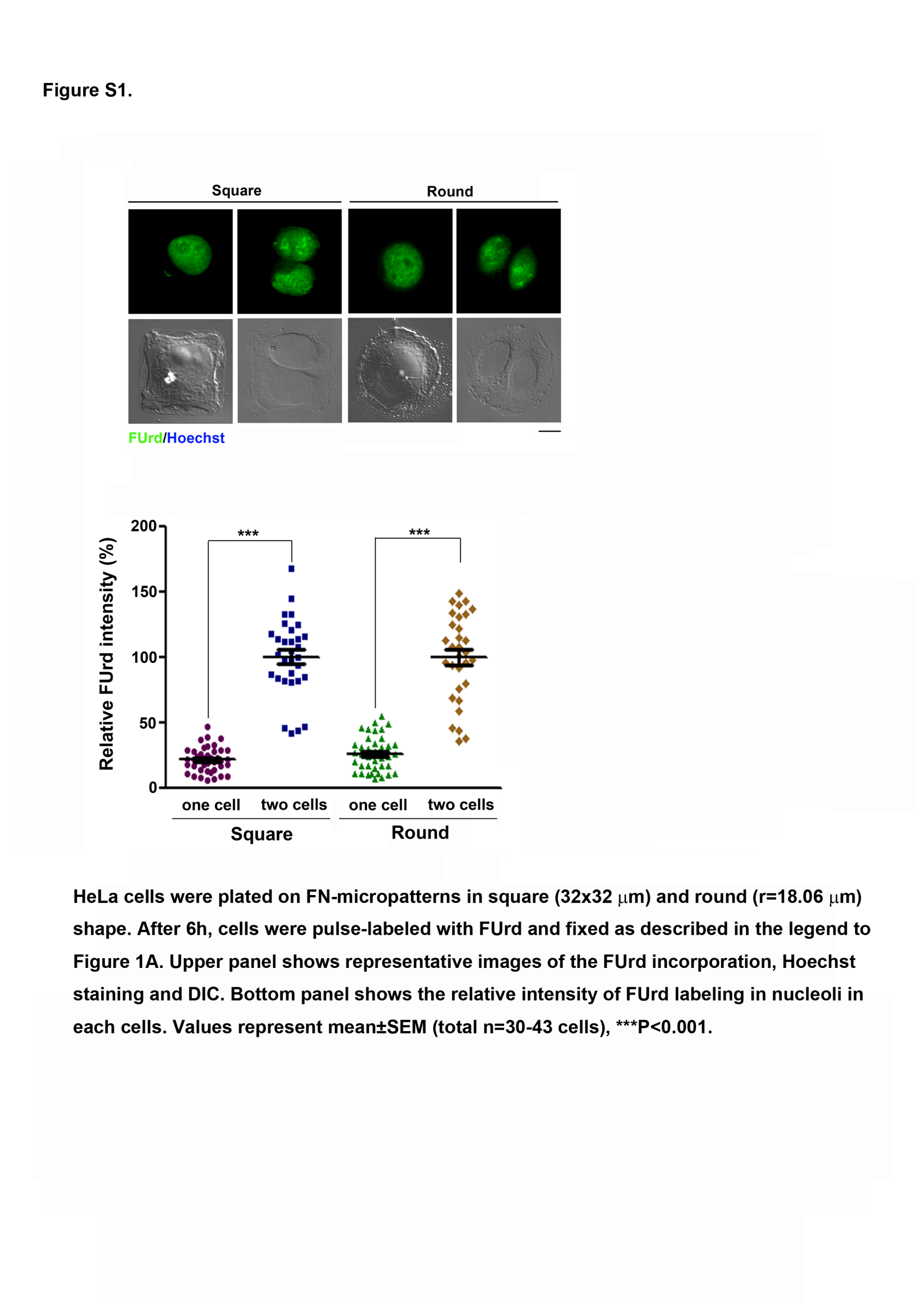
**

**Figure S1.**

HeLa cells were plated on FN-micropatterns in square (32x32 μm) and round (r=18.06 μm) shape. After 6h, cells were pulse-labeled with FUrd and fixed as described in the legend to Figure 1A. Upper panel shows representative images of the FUrd incorporation, Hoechst staining and DIC. Bottom panel shows the relative intensity of FUrd labeling in nucleoli in each cells. Values represent mean±SEM (total n=30-43 cells), ****P*<0.001.

**Supplementary Figure 2**

**
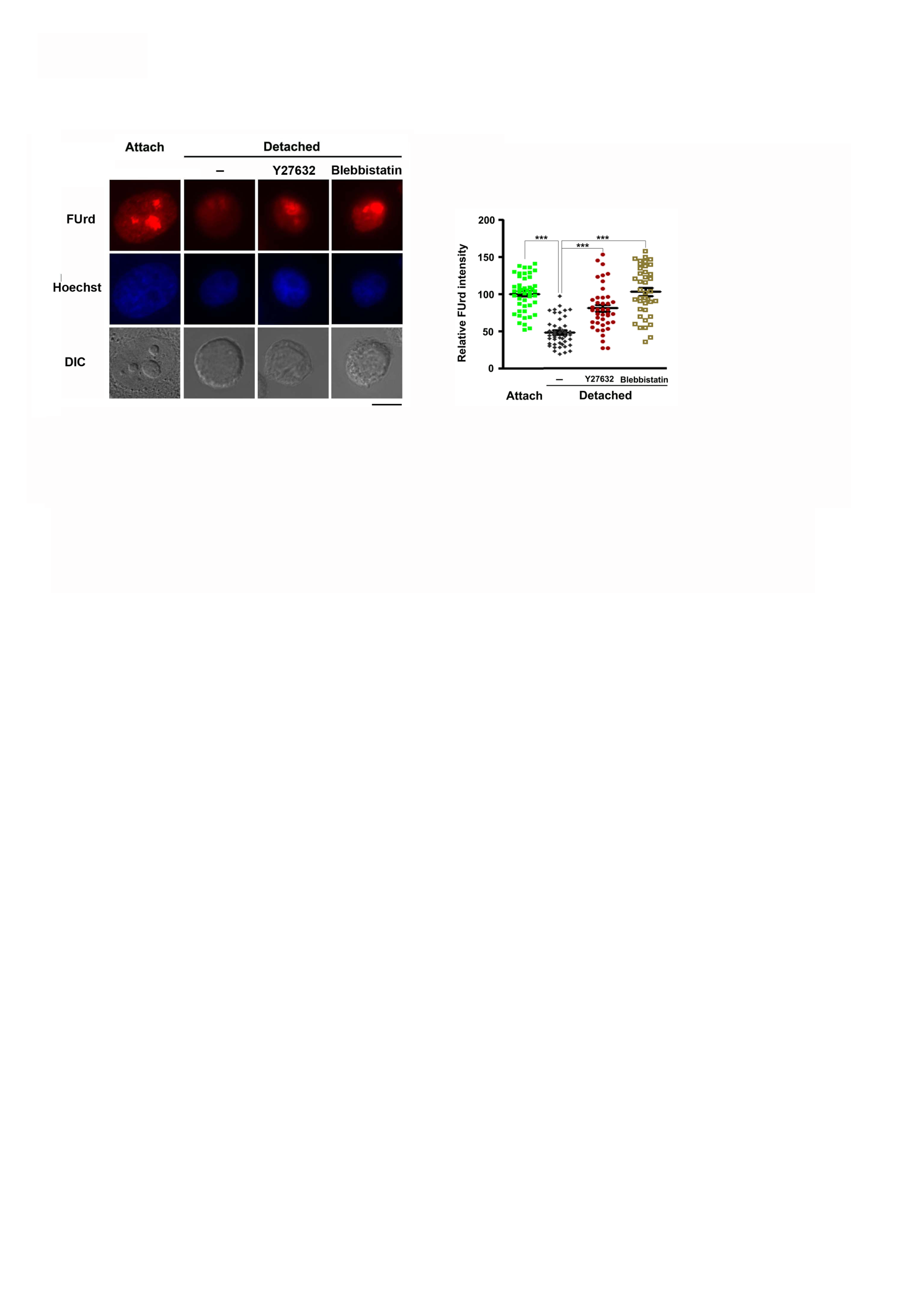
**

**Figure S2.**

HeLa cells were trypsinzed to get rounding shape, followed by plating onto poly-lysine coated plate in the presence of Y27632 or TSA as indicated for 10 min. These cells were then pulse-labeled with FUrd and fixed as described in the legend to Figure 1. Left panel shows representative images of the FUrd incorporation, Hoechst staining and DIC. Right panel shows the relative intensityof FUrd labeling in nucleoli in each cells. The mean of anti-BrdU antibody fluorescence in unconstrained cells was set to 100% (total n>50). Values represent mean±SEM. ****P*<0.001.
